# The emerging roles of vacuolar-type ATPase-dependent Lysosomal acidification in neurodegenerative diseases

**DOI:** 10.1186/s40035-020-00196-0

**Published:** 2020-05-11

**Authors:** Qiaoyun Song, Bo Meng, Haidong Xu, Zixu Mao

**Affiliations:** 1 Department of Pharmacology and Chemical Biology, Emory University School of Medicine, Atlanta, GA 30322 USA; 2 Department of Reproductive Genetics, Hebei General Hospital, Shijiazhuang, Hebei Province 050051 People’s Republic of China; 3 Department of Neurology, Emory University School of Medicine, Atlanta, GA 30322 USA

**Keywords:** Vacuolar-type ATPase, Lysosomal acidification, Neurodegeneration, Autophagy

## Abstract

**Background:**

Lysosomes digest extracellular material from the endocytic pathway and intracellular material from the autophagic pathway. This process is performed by the resident hydrolytic enzymes activated by the highly acidic pH within the lysosomal lumen. Lysosome pH gradients are mainly maintained by the vacuolar (H^+^) ATPase (or V-ATPase), which pumps protons into lysosomal lumen by consuming ATP. Dysfunction of V-ATPase affects lysosomal acidification, which disrupts the clearance of substrates and leads to many disorders, including neurodegenerative diseases.

**Main body:**

As a large multi-subunit complex, the V-ATPase is composed of an integral membrane V0 domain involved in proton translocation and a peripheral V1 domain catalyzing ATP hydrolysis. The canonical functions of V-ATPase rely on its H^+^-pumping ability in multiple vesicle organelles to regulate endocytic traffic, protein processing and degradation, synaptic vesicle loading, and coupled transport. The other non-canonical effects of the V-ATPase that are not readily attributable to its proton-pumping activity include membrane fusion, pH sensing, amino-acid-induced activation of mTORC1, and scaffolding for protein-protein interaction. In response to various stimuli, V-ATPase complex can reversibly dissociate into V1 and V0 domains and thus close ATP-dependent proton transport. Dysregulation of pH and lysosomal dysfunction have been linked to many human diseases, including neurodegenerative disorders such as Alzheimer disease, Parkinson’s disease, amyotrophic lateral sclerosis as well as neurodegenerative lysosomal storage disorders.

**Conclusion:**

V-ATPase complex is a universal proton pump and plays an important role in lysosome acidification in all types of cells. Since V-ATPase dysfunction contributes to the pathogenesis of multiple neurodegenerative diseases, further understanding the mechanisms that regulate the canonical and non-canonical functions of V-ATPase will reveal molecular details of disease process and help assess V-ATPase or molecules related to its regulation as therapeutic targets.

## Background

Lysosomes are the primary degradative compartment of the cell that executes the final destruction and recycling of organelles and molecules. Extracellular materials are delivered to the lysosomes via the endocytic pathway while intracellular materials reach the organelle by the autophagic process [1]. Since the discovery of lysosome by Belgian biochemist Christian de Duve [2], significant progress has been made about the function of the organelle. We now know that lysosomes have diverse functions including turnover of cellular molecules, nutrient sensing, regulation of receptor distribution, antigen presentation, and response to cellular stress etc. [3–5].

The degradative function of the lysosomes is carried out by the resident hydrolytic enzymes within their lumen. These enzymes are activated by highly acidic pH (less than pH 5.0) in the luminal compartment. The acidic pH is primarily mainly maintained by a proton pump, the V-type proton ATPase (V-ATPase), which pumps protons into the lysosomal lumen, a process that requires the consumption of ATP [6]. Dysregulated acidification and increased intraluminal pH suppress the activities of various enzymes in lysosome and disrupt the clearance of various substrates including protein aggregates. Thus, it is not surprising that conditions impairing lysosomal acidification may contribute to a range of diseases, many of which are severe or life threatening. The most well-known diseases related to lysosomes are lysosomal storage disorders (LSDs) [7], a family of disorders caused by inherited gene mutations of lysosomal and non-lysosomal proteins, including the resident hydrolytic enzymes inside the lysosome [8]. The deficiencies of those lysosomal enzymes lead to defective processing of substrates critical for cell survival and the abnormal storage of macromolecular substrates that can cause further lysosomal damage [8]. The resulting abnormal accumulation of protein aggregates are the hallmarks of multiple major neurodegenerative diseases including Alzheimer’s Disease (AD) and Parkinson’s Disease (PD) etc. [9–12]. In this review, we will first introduce the general features of lysosome in neurons, describe the structure and functions of V-ATPase, and highlight the evidence linking deficiencies of V-ATPase components and neurodegenerative diseases.

## Main text

### Lysosome function

Lysosomes degrade a broad range of substrates including DNA, RNA, proteins, and lipid. The size of those substrates varies from single molecule to a whole organelle. Lysosomes, as the final site of destruction, accept and degrade substrate proteins from several distinct processes, including the secretory, endocytic and autophagic pathways. For example, excess secretory granules containing insulin could fuse with lysosomes through a process called crinophagy in which the secretory proteins located in the secretory vesicles are degraded in β-cell [13, 14]. During developmentally programmed crinophagy in Drosophila, the excessive secretory glue granules fuse with lysosomes, and the degradation of secretory material depends on the activity of the Uvrag-containing Vps34 lipid kinase complex and the V-ATPase proton (H^+^) pump [15]. In eukaryotic cells, extracellular macromolecules can be taken up by cells through clathrin-mediated endocytosis. The extracellular macromolecules or ligands bind to cell surface receptors that are concentrated in clathrin-coated pits. The pits then bud from the plasma membrane to form intracellular clathrin-coated vesicles, which are sorted by the early endosome system. Substrates are finally delivered to the lysosomes for degradation [16]. This process regulates the interaction of cells with their environment and provides cells with energy sources such as amino acids, carbohydrates, and lipids as well as substrates utilized for biosynthesis [17]. Endocytic and lysosomal pathway is fundamental to neurotransmission, signal transduction and the regulation of many plasma membrane activities [16, 18]. In contrast, autophagy targets intracellular cytosolic components such as long-lived proteins, protein aggregates, stress RNA granules, and organelles including mitochondria for degradation by lysosomes, and is essential for maintaining cellular homeostasis. There are three defined forms of autophagy, macroautophagy, microautophagy, and chaperone-mediated autophagy (CMA) [13, 19, 20]. Macroautophagy involves wrapping the substrates in the cytosolic compartment into double-membrane vesicles named autophagosomes, transporting autophagosomes and fusing them with lysosomes for degradation [21]. Microautophagy is less well characterized but involves the internalization of cytosolic substrate proteins through invagination of the lysosomal membrane [13]. Microautophagy-like process that delivers soluble cytosolic proteins to the late endosomes/multivesicular bodies (MVBs) has been reported [22]. CMA is a unique form of autophagy that selectively degrades individual proteins. This involves the binding of chaperone Hsc70 to the KFERQ-like pentapeptide motif in substrate proteins [23], interaction of Hsc70/substrate complex with the lysosome-associated membrane protein type 2A (LAMP-2A) on the lysosome, and the subsequent translocation of the substrate proteins into the lysosomal lumen for degradation [24, 25].

Lysosomes need to maintain a highly acidic pH in its lumen in order to successfully perform its digestive function and to drive efflux of digested materials [6]. The acidic environment of the lysosome is critical not only for the function of lysosomes themselves but also for many cellular processes related to lysosomes. One of the most important roles involving the acidic pH is to guarantee the delivery of various enzymes to lysosomes and their proper maturation inside the organelle. The newly synthesized enzymes such as pro-form of cathepsin D are first transported to the late endosomes via a series of steps and the acidic environment of the endosomes is needed for its dissociation from mannose-6-phosphate receptor involved in its proper transportation [26]. Maturation of lysosomal enzymes, which involves sequential cleavage of immature pro-forms to mature forms of hydrolases, depends on the acidification of lysosome [27]. Similarly, normal dissociation of cargoes from their carrier proteins, which is essential for many cellular processes, also requires the acidic environment of lysosome. For example, the dissociation of cholesterol and other lipids from their receptors occurs inside the acidic environment of lysosome [28]. Ferritin is a cytosolic protein that stores excess iron and protects cells from iron toxicity [29]. Lysosomal acidification is crucial for the extraction of iron from ferritin for its degradation in lysosomes [30]. Inhibition of lysosomal acidification triggers cellular iron deficiency, which can cause neuronal stress by impairing mitochondrial function and triggering inflammation in cultured primary neurons [31]. The lysosomes constantly fuse with other organelles including endosomes and autophagsomes. The endosomal and lysosomal lumen pH varies between a range of 4.5 to 6.5 due to the activity of the ATP-dependent proton pumps present on their membrane. This unique acidic environment is for the sequential activation of hydrolases, uncoupling ligands from receptors, inactivation of microbicidal factors, cargo transportation, membrane trafficking, and efficient degradation of various substrates [9, 32]. Dysregulation of the endosomal/autophagosome-lysosomal system has been implicated in several human diseases including neurodegenerative diseases (discuss later) as well as atherosclerosis [33].

### Lysosomes in neurons

The brain has abundant amount of lysosomes and this lysosomal network offers neurons a constitutively and highly efficient degradative system [34]. Lysosome-mediated continuous clearance of cellular proteins through autophagy is important for maintaining neuronal homeostasis. This basal process is essential since deficiency of autophagic genes causes the accumulation of intracellular proteins and inclusion bodies, and ultimately neurodegeneration [35, 36]. Disruption of cathepsin- mediated proteolysis leads to marked accumulation of autophagosome in the brain, which resembles the vesicles in the brain of AD patients and model mice [34]. Since neurons are postmitotic and polarized structurally, they are particularly sensitive to the impairment of lysosomal function [37]. The lysosomal system in neurons is easily affected when lysosomal hydrolysis is impaired, leading to rapid accumulation of the materials bound for degradation [38–40]. Thus, abnormal or defective lysosomal function affects many aspects of neuronal processes and has been identified as a critical factor contributing to or even driving the pathogenic process of many neurodegenerative diseases, including AD, PD, and Huntington’s disease (HD) etc. [41–45]. It’s worth noting that multiple lysosomal functions including its proteolytic activity decline with aging [46]. Aging itself is the biggest risk factor for AD and PD. Therefore, it is possible that part of the increased risk for AD and PD associated with aging is due to the decline of lysosomal activity and the consequential accumulation of toxic materials including damaged organelles and proteins. Thus, aging and genetic defects converge on the lysosomes, whose dysfunction may participate in not only the initiation but also the acceleration of diverse neurodegenerative processes.

The involvement of lysosomes in various neuronal processes requires and depends on their ability to move throughout the cytoplasm [47]. Indeed, it has long been recognized that lysosomes in neurons are not distributed evenly but in a polarized manner. They are particularly abundant in neuronal cell body. Fewer are present in dendrites and they are even more rare in axons [48–50]. This indicates that lysosomes in neurons are separated into different compartments to match the degradative needs of various compartments and a large portion of the final degradation mainly occurs in the cell body. Neurons are challenged to deliver unwanted components from the periphery compartments to the cell body along the long distance of axons or dendrites. In support of this, disruption of the transportation system in neurons with microtubules depolymerization compounds leads clearly to rapid autophagic vacuoles accumulation [34]. Such polarized distribution of lysosomes suggests that neurons have different mechanisms to control the movement of lysosomes in neurons [51–54].

### V-ATPase and its structure

There are two related families of (H^+^) ATPases: the family of F-type proton-translocating ATPases (F-ATPases) and the family of vacuolar (H^+^) ATPase (V-ATPase). The two families of ATPase are evolutionarily related and share structural similarities [55]. One enzymatic difference between the two families of ATPase is that F-ATPase is involved in synthesizing as well as hydrolyzing ATP [56] while V-ATPase seems to function mainly in proton pumping by consuming ATP [57]. The F-ATPases are found in bacterial plasma membrane, mitochondrial inner membrane and in chloroplast thylakoid membrane [58, 59]. In addition to being present on the plasma membrane of certain eukaryotic cells, V-ATPase is widely distributed on the membrane of various subcellular organelles, such as lysosome, Golgi, and secretory granules. Each organelle maintains a characteristic internal pH, which is essential for facilitating organelle function, such as endocytosis, exocytosis, membrane flow and substance transport. The V-ATPase is the main driving force of the acid pH of the vacuolar system in eukaryotic cells. It pumps protons into the lumen of an organelle using the energy generated by ATP hydrolysis.

**Fig. 1:**
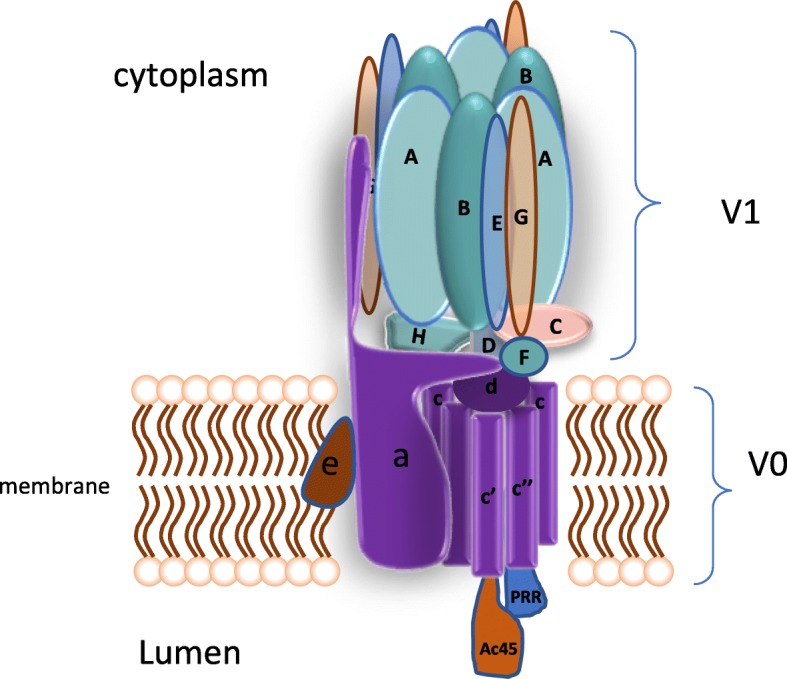
Structure of V-ATPases. V-ATPase is composed of multiple core subunits and two accessory proteins. The cytosolic V1 domain includes eight different subunits (A, B, C, D, E, F, G, and H) and the integral V0 domain contains six different subunits (a, c, c’, c”, d, and e). The hypothetic positions of the ATP6AP1/Ac45 and ATP6AP2/PRR are included

The V-ATPases are large multi-subunit complexes composed of two domains: a membrane integral V0 sector involved in proton translocation and a peripheral V1 domain catalyzing ATP hydrolysis. As shown at Fig. 1, the integral V0 domain is a 260 kDa complex containing six different subunits (a, c, c’, c”, d, and e) whereas the V1 domain is a 640 kDa complex including eight different subunits (A, B, C, D, E, F, G, and H) [60, 61]. In higher eukaryotes, several different H^+^-ATPase subunits have multiple isoforms encoded by separate genes that are located throughout the genome with differing tissue expression patterns [62–64]. For example, there are two isoforms for the B, E, d, and e subunits; three for the C and G subunits; and four for the a subunit. Some of the isoforms have different expression patterns in various tissues. For example, the d1 subunit is ubiquitously expressed while the d2 homolog is expressed only in the kidney, osteoclast and lung [65]. Similarly, the G1 isoform is expressed ubiquitously while G2 and G3 isoforms are found mainly in neuronal tissue and kidney [66]. Some subunits are encoded through splice variants, such as the a, d, e, C, G and H subunits [67–71]. This complexity leads to different possible permutations of subunit structure in individual proton pumps and unique subunit identities of pumps at different locations, which may allow the regulation of V-ATPase in a cell type and subcellular compartment specific manner. The peripheral domain and the integral domain are assembled separately and brought together into a functional proton pump at the required organelles [72]. In neuronal and neuroendocrine cells, the V-ATPase is equipped with the accessory subunits ATP6AP1 and ATP6AP2. ATP6AP1, also known as Ac45, was first identified as the accessory subunit of V-ATPase in chromaffin granules [73]. ATP6AP1 functions to guide the V-ATPase to certain subcellular compartments such as neuroendocrine regulated secretory vesicles and regulates their activity [74], the intragranular pH and Ca^2+^-dependent exocytotic membrane fusion [75]. ATP6AP2 was first identified as the C-terminal fragment of the (pro) renin receptor (PRR) for renin and prorenin [76]. Ablation of PRR in cardiomyocytes reveals that PRR is an integral component for the stability and assembly of V0 subunits [77]. ATP6AP2 is a key accessory protein for V-ATPase functions in the CNS and essential for stem cell self-renewal and neuronal survival [78].

### Regulation of V-ATPase

V-ATPase’s proton pumping activity plays a vital role in numerous essential cellular processes such as pH and ion homeostasis, protein trafficking, autophagy, endocytosis, signaling, and neurotransmitter release [17, 79–82]. The activity of V-ATPase is regulated by multiple mechanisms. First, the vesicle V-ATPase activity is regulated by the formation of intramolecular disulfide bonds and this is modulated by ATP or high ionic strength [83]. Studies have shown that formation and cleavage of a disulfide bond between cysteine residues at or near the catalytic site of the enzyme [84], such as the intramolecular disulfide bond formed between cysteine 254 and cysteine 532 in subunit A of the bovine V-ATPase, result in the inactivation of the enzyme [85]. A study of plant V-ATPase shows that E subunit can also form intramolecular disulfide bonds that change its conformation [86].

Regulation of V-ATPase can be achieved by the control of the abundance of V-ATPase or some of its subunits as well as their localization on the membrane [87]. For example, the level of E1 subunit increases in the medulla of kidneys from acetazolamide-treated rats [88]. The cytoplasmic membrane and intracellular vesicles containing high-density V-ATPase membrane may fuse, which leads to the adjustment of proton pump density [89]. Several specialized cells such as interdental cells of the ear, epithelial cells of the nose, vision or proximal tubule of kidney also express V-ATPase at their plasma membrane, which is involved in extracellular pH regulation [90–93]. The localization of V-ATPase to plasma membrane is controlled through reversible exocytosis and endocytosis of vesicles that contain a high density of V-ATPase [94].

In response to various stimuli, V-ATPase complexes can reversibly dissociate into V1 and V0 domains and thus close ATP-dependent proton transport. In yeast, reversible dissociation occurs rapidly in response to glucose depletion. Glucose depletion even for as little as 5 min causes dissociation of approximately 70% of the assembled V-ATPase enzyme complex into separate V1 and V0 subcomplexes. Restoration of glucose induces rapid and efficient reassembly of the enzyme from the previously synthesized subcomplexes [95]. Assembly requires the glycolytic enzyme aldolase and RAVE complex (regulator of H^+^-ATPase of vacuolar and endosomal membranes) [96, 97], while dissociation, which is independent of new protein synthesis, requires intact microtubules [98]. Rather than leading to disassembly, studies in mammalian cells have demonstrated that acute glucose depletion can increase V-ATPase assembly and activity through AMPK and PI3K-Akt signaling pathway [99, 100]. Amino acid starvation has been shown to promote V-ATPase assembly and re-addition of amino acid reverses this effect [101]. These findings highlight the importance of reversible dissociation of V1-V0 complexes as a common mechanism in efficiently regulating V-ATPase in response to signals.

V-ATPase acidifies the newly formed synaptic vesicles to generate a proton electrochemical gradient that drives neurotransmitter loading and clathrin-mediated endocytosis [102]. Recently, Milosevic et al. proposed a model of regulation of synaptic vesicle V-ATPase. It is thought that the activity of V-ATPase in the clathrin-coated vesicles (CCVs) is inhibited by the clathrin coat. When the coat is removed from CCVs, V-ATPase becomes functional and acidifies the uncoated vesicles [103]. This regulation of V-ATPase activity may be an important mechanism for the proper timing of synaptic vesicles refilling.

### Role of V-ATPase in the nervous system

**Fig. 2:**
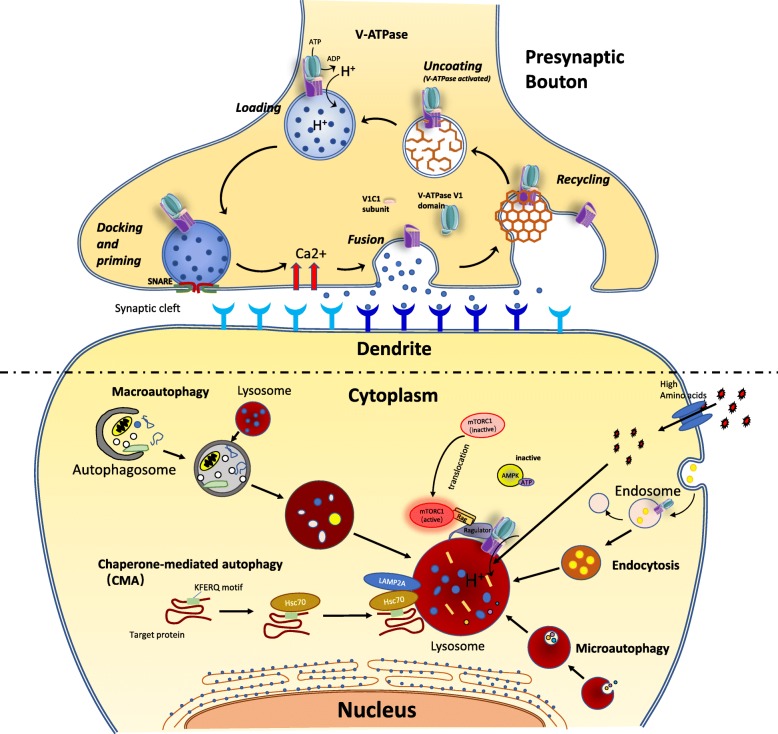
Function of V-ATPase in the nervous system. In presynaptic bouton, V-ATPase is responsible for generating the H^+^-electrochemical gradient in synaptic vesicles, which drives the refilling of newly formed synaptic vesicles with neurotransmitter. Synaptic vesicle V-ATPase also participates in the step of fusion. Relying on its H^+^-pumping ability, V-ATPase modulates multiple cellular activities including endosome maturation and trafficking, protein processing and degradation via different autophagic pathways in multiple vesicle organelles such as lysosome and endosome. The acidic environment of the lysosomes is critical for not only the function of lysosomes but also many cellular processes related to lysosomes. V-ATPase is also involved in pH sensing, nutrient signaling, and scaffold for protein-protein interactions

V-ATPases have multiple cellular functions at different intracellular sites. The main functions of V-ATPase rely on its H^+^-pumping ability in multiple membrane organelles such as lysosome, endosome, and synaptic vesicles. Through this, V-ATPase modulates a range of cellular activities including endocytic traffic, protein processing and degradation, synaptic vesicles loading and coupled transport [82, 104–106]. There are also other non-canonical effects of the V-ATPase that are not readily attributable to its proton-moving activity. This includes membrane fusion, pH sensing, amino-acid-induced activation of mammalian target of rapamycin complex1 (mTORC1), and scaffold for protein-protein interactions, etc. [5, 107, 108]. Some of the main functions of V-ATPase in neurons are summarized in Fig. 2.

#### Synaptic vesicle loading and coupled transport

It is known that V-ATPase generates the H^+^-electrochemical gradient in synaptic vesicles and this gradient is necessary to drive the refilling of newly formed synaptic vesicles with neurotransmitter [109]. Some vesicular transporters such as monoamine transporters utilize the H^+^-concentration gradient (ΔpH) whereas glutamate transporters rely primarily on the electrical potential (Δψ) generated by the V-ATPase [110]. The uptake of the positively charged acetylcholine and γ-aminobutyric acid (GABA) rely on both ΔpH and Δψ. The synaptic vesicles with neurotransmitter loaded undergo reversible tethering at the plasma membrane where they fuse and release the neurotransmitters into the synaptic space. Synaptic vesicle V-ATPase also participates in the step of fusion (discussed below).

#### Endosome maturation and trafficking

Defects in membrane trafficking and degradation result in the accumulation of undegraded proteins due to aberrant endosomal sorting, lysosomal degradation, or autophagy. Alterations of membrane trafficking are known to occur in AD, other tauopathies, PD, peripheral neuropathies, lysosomal storage disorders and PolyQ diseases [111]. The luminal pH is an important aspect of endosome maturation and function. Blocking V-ATPase function by pharmacological inhibitors or by siRNA leads to the accumulation of materials in the early endosomes and inhibits subsequent endocytosis [104, 105]. V-ATPase deficiency results in the formation of large hybrid organelles containing markers of immature lysosomes, granules, and autophagy, affecting the pathways of degradation and secretion [112]. These studies suggest that normal pH, which is dependent on V-ATPase-mediated acidification, is essential for sorting function of the endosomal system and subsequent endosome-lysosome processing.

#### pH acidification and sensing

Tight control of pH homeostasis is essential for a vast number of physiological processes of several organelles. For example, the pH of early endosomes (EEs) is between 6.8–6.1 range and the pH of late endosomes (LEs) is around 5.5. pH inside lysosome reaches the values of 4.5–4.7. The acidic pH of endocytic organelles not only provides the environment for the activation of the degradative enzymes but also is essential for membrane trafficking and sorting endosomes to promote the dissociation of internalized ligand–receptor complexes during receptor-mediated endocytosis. This process also plays a role in the inactivation of internalized pathogens [60, 113]. Recently, V-ATPase has been reported to serve as a pH sensor of endosomes in renal tubule cells [107]. The study shows that the small GTPase Arf6 and its cognate GDP/GTP exchange factor (GEF) ARNO (ADP-ribosylation factor nucleotide site opener) directly interact with the c-subunit and the a2-subunit of V-ATPase, respectively. The interaction of a2-isoform and ARNO is dependent on the intra-endosomal acidification, and disruption of this interaction inhibits endocytosis. These results suggest that the subunits of V-ATPase may perform additional functions in a luminal pH dependent manner.

#### Membrane fusion

Recent studies suggest that the transmembrane V0 domain of V-ATPase promotes membrane fusion independent of its role in acidification. The direct involvement of V0 domain of the V-ATPase in the process of membrane fusion comes from the studies of fusion in yeast [114]. Mutation of genes that encode subunits of the V0 domain or using inhibitory antibodies has a more significant effect on yeast vacuolar fusion than inhibition of V1 domain [115]. SNARE (soluble N-ethylmaleimide- sensitive factor attachment protein receptor) mediates the attachment step in the membrane fusion of vesicles. The V0 is involved in forming complexes between opposing membranes. After the trans-SNARE pairing, V0 trans-complex forms dependent on both Rab-GTPase Ypt7 and calmodulin [114]. Several studies from the nervous system and cells also reveal that V-ATPase is required for membrane fusion during exocytosis of synaptic and secretory vesicles [108, 116, 117]. In *Drosophila melanogaster*, a neuronal isoform of a subunit of V0 component, vha 100–1, is shown to colocalize with synaptic vesicles and active zones. Mutation of vha 100–1 results in accumulation of synaptic vesicles in synaptic terminals with normal levels of neurotransmitter loading [108], indicating the blocking of synaptic vesicle fusion with the presynaptic membrane. Mutation in the V0a subunit of *Caenorhabditis elegans* blocks the secretion of Hedgehod-related proteins through apical secretion without affecting vesicle acidification [118]. Similarly, deletion of the a3 isoform of V-ATPase impairs secretion of insulin from pancreatic β-cell without significant alternation in the pH of the secretory vesicle [117]. It should be noted that the role of V0 domain in membrane fusion has not been completely resolved. Although the above results suggest that V-ATPase has a role in membrane fusion independent of acidification, the exact mechanism by which V-ATPase promotes membrane fusion needs further clarification.

#### Nutrient signaling

mTOR is a serine/threonine kinase that belongs to the phosphoinositide kinase-related family. mTOR integrates signals from growth factors and amino acid availability to control cell growth. This functional enzyme is present in two distinct complexes: mTOR complexes (mTORC1 and mTORC2), both of which are characterized by different protein partners and specific substrates [119]. V-ATPase is necessary for the activation of nutrient signaling from mTORC1 and AMPK [17, 120, 121]. Amino acids promote the translocation of mTORC1 to the lysosomal surface, where it is activated [122]. The V-ATPase is necessary for amino acids to activate mTORC1 by interacting with the Ragulator, a scaffolding complex that anchors the Rag GTPase to the lysosome [17]. V-ATPase-Ragulator complex on late endosomes or lysosomes is also required for the activation of resident AMPK present on these two organelles, thus providing a switch between catabolism and anabolism [120]. It has been shown that inhibition of V-ATPase with its inhibitors Bafilomycin A1 or Concanamycin A increases the luminal concentrations of most metabolites but has no effect on the majority of essential amino acids in the lysosomes. But nutrient starvation-mediated inhibition of mTOR reduces the lysosomal efflux of most essential amino acids [123]. These results suggest that V-ATPase- and mTOR-dependent mechanisms exist for controlling lysosomal flux of metabolites.

### Dysfunction of V-ATPase-dependent lysosomal acidification in neurodegenerative diseases

As our knowledge of the lysosome as a multifunctional organelle in cellular clearance, signaling and energy metabolism progresses, the importance of its pH homeostasis becomes increasingly recognized [124]. Dysregulation of pH and lysosomal dysfunction are being linked to the congenital CNS diseases such as Renal tubular acidosis with deafness [125, 126], early-onset CNS diseases such as X-linked Parkinson Disease with Spasticity (XPDS) [127, 128], Wolfram syndrome [129–131], and adult-onset neurodegenerative disorders such as AD, PD, and amyotrophic lateral sclerosis [132, 133]. Here we focus on the LSD, PD and AD.

#### Neurodegenerative lysosomal storage disorders

In eukaryotes, lysosomes are the main organelles for intracellular digestion [2]. It contains > 50 hydrolases that require an acidic pH for optimal degradation [134]. It has been reported that dysregulation of lysosomal acidification contributes to pathogenesis in virtually all LSDs [135, 136], which include neuronal ceroid lipofuscinosis (NCL), also known as Batten’s disease. This is a group of the most prevalent neurodegenerative LSDs caused by mutations in more than 13 different genes called the CLNs (ceroid lipofuscinosis neuronal) [137], Niemann-Pick type C (NPC), and mucolipidosis type IV (MLIV) [138]. It has been reported that inactivating mutations in the CLN1 gene, which encodes palmitoyl-protein thioesterase-1(PPT1) can cause infant NCL (INCL) [139, 140], a devastating NLSD. In neurons of Cln1−/− INCL model mice, lack of PPT1 activity causes V0a1 misrouted to plasma membrane, preventing its interaction with AP-3, which is required for its transport from the sorting endosome to the late endosomal/lysosomal membrane. This impairs lysosomal V-ATPase activity, thereby dysregulating lysosomal acidification [141]. These findings reveal a role of Cln1 in regulating lysosomal targeting of V0a1 and suggest that dysregulation of lysosomal acidification caused by varying factors adversely affecting V-ATPase function may be common in other LSDs and neurodegenerative diseases.

#### Parkinson’s disease

PD is caused by the interaction of various genetic factors, environmental factors, and the process of aging [142]. Many of the genetic factors, such as Leucine-rich repeat kinase 2 (LRRK2), α-synuclein (SNCA), Parkin RBR E3 ubiquitin protein ligase (PARK2), Parkinson protein 7 (PARK7), PTEN induced putative kinase 1 (PINK1), scavenger receptor class B, member 2 (SCARB2), etc., are involved in the autophagy-lysosome pathway [143–145]. Notably, Glucocerebrosidase (GBA) gene, which encodes a lysosomal hydrolase glucocerebrosidase (GCase) and whose mutation causes the lysosomal storage disorder Gauchers disease, has recently been shown to be a genetic risk factor for PD when mutated [146]. On the one hand, lysosomes are responsible for the elimination of several PD related toxic factors, such as misfolded proteins or aggregation-prone α-synuclein, and old or damaged mitochondria. Lysosomal failure leads to aberrant accumulation of these toxic materials, which is the molecular hallmark of pathogenic events in PD. On the other hand, many of the genetic risk factors associated with PD often adversely affect V-ATPase and lysosomes. For example, PC12 cells expressing A53T mutant α-synuclein, but not the wildtype α-synuclein, showed lysosomal de-acidification, measured by staining with the acidotropic dye lysotracker, and disruption of lysosomal function [147, 148]. Mutations of ATP6AP2 impair V-ATPase function and result in lysosomal de-acidification, causing lysosomal system failure in neurons and juvenile-onset Parkinsonism [149, 150]. Patients with exon-skipping mutations in ATP6AP2 show cognitive disorders such as XPDS [127, 128]. Another splice site mutation in ATP6AP2 is linked to X-linked intellectual disability (XLID), epilepsy and parkinsonism [151].

ATP13A2 gene (also known as PARK9) encodes a transmembrane endo−/lysosomal- associated P5-type ATPase. Recent study shows that ATP13A2 regulates endosomal and lysosomal cargo sorting and proteostasis through PI (3,5) P2-mediated scaffolding function [152]. Mutation of ATP13A2 is found in autosomal recessive forms of early-onset Parkinsonism and linked to lysosomal dysfunction [153–155]. Mechanistically, fibroblasts derived from PD patients with loss of ATP13A2 function show impaired lysosomal acidification. This is correlated with a decrease in proteolytic processing by lysosomal enzymes, in lysosome-mediated clearance of autophagosomes, and in degradation of lysosomal substrates [153, 155].

The mutation of LRRK2 is regarded as the most frequent cause of familial PD [156]. LRRK2 mutation results in lysosomal expansion and diminished lysosomal degradation of substrates. There is evidence supporting LRRK2 and V-ATPase interaction. In a *C. elegans* model, mutations of LRRK2 cause an increase in V-ATPase subunits as well as the level of V-ATPase modified by 4-hydroxy-2-nonenal (HNE), indicating an increase in oxidative damage to V-ATPase and other proteins as observed in the cerebral cortex in the case of sporadic PD [157, 158]. Recently, LRRK2 is found to associate directly with the a1 subunit of V-ATPase. This interaction is abolished by the LRRK2 R1441C mutation, leading to a decrease in a1 protein and its cellular mis-localization [159].

In addition to genetic factors, many environmental neurotoxic agents, such as methamphetamine, rotenone and MPP^+^, promote PD and PD-like symptoms via impairing lysosomal acidification and lysosomal activity [160–166]. Therefore, dysfunction of lysosomal acidification plays a general role in genetic, sporadic, and toxin-induced forms of Parkinsonism.

#### Alzheimer’s disease

The aggregation of Aβ, the main component of amyloid plaques in AD, is regarded as one of the key pathogenic factors contributing to AD [167]. Aβ is processed from amyloid precursor protein (APP) mainly in the late endosomes into sAPPβ and α-CTF by the action of beta secretase-1 (BACE1), whose peak activity is at pH 4.5 [168]. sAPPβ is subsequently processed into As by γ-secretase. Either an increase in Aβ production or a decrease in its degradation can lead to the accumulation of Aβ and thus leads to the pathological process of AD [169, 170]. Conditions that cause lysosomal dysfunction may prolong the residence time of APP in the acidic late endosomes and thus increase Aβ production. Glial cells participate in the regulation of Aβ level in the brain. Multiple types of brain cells participate in the regulation of Aβ level in the brain [171, 172]. For example, microglia phagocytose Aβ and degrade it via the lysosomal enzymes cathepsin B [173] or endothelin-converting enzymes-2 (ECE-2), a metalloendopeptidase [174]. Several studies suggest that the APP processing machinery may be linked to V-ATPase. Presenilin-1 (PS1) is a component of γ-secretase involved in APP processing and appears to be essential for the targeting of V-ATPase to lysosomes. It has been reported that loss-of-function of PS1 is associated with V-ATPase deficiency, contributing to abnormal cellular Ca^2+^ homeostasis and lysosomal/autophagy dysfunction [175]. Moreover, N-glycosylation of the V0a1 subunit, which is essential for its efficient ER-to-lysosome delivery, requires the selective binding of PS1 holoprotein to the un-glycosylated V0a1 and the Sec61α in the translocon and OST (oligosaccharyltransferase) complex [10]. Sec61αis a subunit of translocon. It is speculated that PS1 binding to translocon facilitates the presentation of the V0a1 subunit to the OST complex, thus promoting its glycosylation and export to lysosomes. In 5 × FAD mice, in which Aβ is over-produced in part due to dysfunctional PS1, the lysosomal acidification is also impaired by a decrease in N-glycoyslation of the V-ATPase subunit V0a1 and reduced cathepsin D activity. Administration of a competitive GSK-3 inhibitor, L803-mts, restores lysosomal acidification in 5 × FAD brains, enhances the clearance of Aβ, and ameliorates the cognitive dysfunction [176]. However, a subsequent study by Zhang et al. failed to observe the alterations in the N-glycosylation of V0a1 in mouse embryonic fibroblasts (MEFs) deficient in PS1 and PS2 [177]. Nor did it find evidence that the turnover of autophagic substrates, vesicle pH or lysosome function is altered in cells lacking PS1 and PS2. Thus, the precise link between V0a1 and PS1 remains to be clarified.

In addition to AD, V-ATPase has also been linked to cognitive function. For example, altered splicing and conditional knockout of the ATP6AP2 gene in mouse and fly have been shown to cause cognitive impairment and other congenital disorders associated with neurodegeneration [178, 179]. Pathologically, ATP6AP2 conditional knockdown in mouse and fly results in presynaptic transmission defects and abnormal changes of both the number and morphology in synapses. Molecularly, lack of ATP6AP2 also triggers autophagy defects and axonal transport alterations. Consistent with these animal studies, patients with ATP6AP1/Ac45 deficiency display neurocognitive abnormalities as well as immunodeficiency phenotype associated with hypogammaglobulinemia and hepatopathy [180].

We summarize the V-ATPase subunits and their related genes dysfunction in congenital, early-onset, and late-onset CNS diseases in Table 1.

**Table 1:** Summary of changes of V-ATPase and neurodegenerative diseases

Gene	Diseases	Species/model	Pathological Mechanisms	Reference
V0a1	Alzheimer Disease	5 × FAD mice	decrease of N-glycosylation of V0a1	[176]
		PS/APP mice	decrease of mature V0a1 in the lysosomal fraction	[181]
V0a2	Autosomal recessive cutis laxa typeII/Wrinkly Skin Syndrome	human	abnormal glycosylation of serum proteins (CDG-II) and impairment of Golgi trafficking by V0a2 mutation	[182–185]
V0a3	Autosomal recessive osteopetrosis with neurodegeneration	R444L mutant mice	endoplasmic reticulum retention and misprocessing of V0a3 due to R444L mutation	[186]
		human	loss of V0a3 function due to truncation or impaired splicing caused by mutations	[187, 188]
V0a4	Renal Tubular Acidosis with hearing loss	human	mutations	[189]
		V0a4−/− mice	proximal tubule dysfunction with defective endocytic trafficking and accumulation of lysosomal material with V0a4 knockout	[190, 191]
V1B1	Renal Tubular Acidosis with hearing loss	human	mutations	[189]
V1B2	Zimmermann-Laband syndrome	human	impaired complex assembly due to missense mutation	[192]
	Dominant Deafness-Onychodystrophy syndrome	human	c.1516C > T mutation	[193]
	cognitive deficits	ATPV1B2 mutant mice	weaker interaction with the V1E2E and abnormal brain development	[194]
ATP6AP2	X-linked mental retardation and epilepsy	human	impairment of ERK1/2 activation	[127]
	X-linked Parkinson Disease	human	overexpression of a minor splice isoform due to mutation	[128]
	cognitive impairment	ATP6AP2 conditional knockout Drosophila/mice	defects in presynaptic transmission and synapses abnormal caused by conditional knockout	[179]
ATP13A2	Neuronal ceroid lipofuscinosis	human	mutation	[195]
		ATP13A2 ko mice	increased insoluble α-synuclein in the hippocampus	[196]
	Kufor-Rakeb syndrome	ATP13A2 ko mice	increase in gliosis, lipofuscinosis and lysosomal markers; protein aggregation but no α-synuclein abnormalities; selective defects in autophagy	[197]
	Hereditary parkinsonism	human/in vitro	retaintion in the endoplasmic reticulum and degradation by the proteasome due to truncation	[198]
WFS1	Wolfram Syndrome	human	mutation	[130]
		WFS1−/− mice	V1A/V1B instability	[199]
CLN1	Neuronal ceroid lipofuscinoses	human	mutation	[139]
		CLN1−/− mice	misrouting of V-ATPase subunit V0a	[141]

## Conclusion

Although V-ATPase complex is a universal proton pump and plays important roles in lysosome acidification for all cell types, the most profound impact of V-ATPase dysfunction appears to manifest in the central nervous system as neurodegeneration. There is evidence that targeting lysosomal acidification and V-ATPase offers therapeutic benefits [200–203]. For example, studies have demonstrated that FK506 exerts neuroprotective effects by binding with ATP6V1A and induces autophagy. This provides a new and attractive strategy for treating neurodegenerative diseases [204]. Dendrobium nobile Lindl Alkaloids (DNLA) has been used in APP/PS1 mice, which exhibit impaired lysosomal function, and shown to increase the level of V-ATPase A1 subunit and improve learning and memory function in this AD model [205]. In further support of this strategy, low-dose bafilomycin A1 has also been proved to be cytoprotective in part through its maintenance of the autophagy-lysosome pathway [206]. Similarly, the bafilomycin A1-binding subunit ATP6V0C may also serve as a therapeutic target to enhance substrate degradation in age-related neurodegenerative disease [207]. In this regard, it is particularly worth noting that the diversity of subunit isoforms existed for the V-ATPases in different organs and organelles should offer the opportunities for selectively targeting particular V-ATPase complexes that are involved in certain disease processes. Further understanding the molecular details that regulate V-ATPase in the CNS may identify additional and attractive targets for treating neurodegenerative diseases.

## Data Availability

Not applicable.

## Abbreviations

Aβ: amyloid beta
AD: Alzheimer’s Disease
ARNO: ADP- ribosylation factor nucleotide site opener
ALPs: autophagy-lysosomal pathways
ALS: amyotrophic lateral sclerosis
AP: autophagosomes
APP: amyloid precursor protein
BACE1: beta secretase-1
CLNs: ceroid lipofuscinosis neuronal
CMA: chaperone-mediated autophagy
CI-M6PR: cation-independent mannose-6-phosphate receptor
DNLA: Dendrobium nobile Lindl Alkaloids
ECE-2: endothelin-converting enzymes-2
EEs: early endosomes
HD: Huntington’s disease
HEK-293 T: human embryonic kidney-293 T
HNE: 4-hydroxy-2-nonenal
INCL: infant NCL
GBA: Glucocerebrosidase
LSDs: lysosomal storage disorders
LEs: late endosomes
LAMP-2A: lysosome-associated membrane protein type 2A
LRRK2: Leucine-rich repeat kinase 2
MEFs: mouse embryonic fibroblasts
MLIV: Mucolipidosis type IV
mTOR: Mammalian target of rapamycin
mTORC1: mechanistic target of rapamycin complex 1
MVBs: multivesicular bodies
NCLs: neuronal ceroid lipofuscinoses
NLSD: Neurodegenerative lysosomal storage disorders
NPC: Niemann-Pick type C
NTs: Neurotransmitters
OST: oligosaccharyltransferase
PARK2: Parkin RBR E3 ubiquitin protein ligase
PARK7: Parkinson protein 7
PD: Parkinson’s disease
PINK1: PTEN induced putative kinase 1
PPT1: palmitoyl-protein thioesterase-1
PS1: presenilin 1
PS2: presenilin 2
RAVE: regulator of H^+^-ATPase of vacuolar and endosomal membranes
SCARB2: scavenger receptor class B, member 2
SNARE: soluble N-ethylmaleimide-sensitive factor attachment protein receptor
SNCA: α-synuclein
V-ATPase: vacuolar-type ATPase
XLID: X-linked intellectual disability
XPDS: X-linked Parkinson’s disease with sputum
